# Transparent Bendable Secondary Zinc-Air Batteries by Controlled Void Ionic Separators

**DOI:** 10.1038/s41598-019-38552-4

**Published:** 2019-02-28

**Authors:** Ohchan Kwon, Ho Jung Hwang, Yunseong Ji, Ok Sung Jeon, Jeong Pil Kim, Chanmin Lee, Yong Gun Shul

**Affiliations:** 1 Department of Chemical and Biomolecular Engineering, Yonsei University, 262 Seongsanno, Seodaemun-gu, Seoul 120-749 South Korea; 2 New energy and battery engineering, Yonsei University, 134 Shinchon-dong, Seodaemoon-ku, Seoul 120-749 Republic of Korea

## Abstract

First ever transparent bendable secondary zinc-air batteries were fabricated. Transparent stainless-steel mesh was utilized as the current collector for the electrodes due to its reliable mechanical stability and electrical conductivity. After which separate methods were used to apply the active redox species. For the preparation of the anode, zinc was loaded by an electroplating process to the mesh. For the cathode, catalyst ink solution was spray coated with an airbrush for desired dimensions. An alkaline gel electrolyte layer was used for the electrolyte. Microscale domain control of the materials becomes a crucial factor for fabricating transparent batteries. As for the presented cell, anionic exchange polymer layer has been uniquely incorporated on to the cathode mesh as the separator which becomes a key procedure in the fabrication process for obtaining the desired optical properties of the battery. The ionic resin is applied in a fashion where controlled voids exist between the openings of the grid which facilitates light passage while guaranteeing electrical insulation between the electrodes. Further analysis correlates the electrode dimensions to the transparency of the system. Recorded average light transmittance is 48.8% in the visible light region and exhibited a maximum power density of 9.77 mW/cm^2^. The produced battery shows both transparent and flexible properties while maintaining a stable discharge/charge operation.

## Introduction

Augmented reality (AR) is a topic of interest that has been gaining significant momentum in the current electronics field^1^. Although in its early stages, AR is perceived as an innovative, facile method offering users with real-time information at the cue of a glance. Most electronics manufactures execute AR technology by displaying the augmented data simultaneously on the display panel where the viewer’s non-augmented perception is captured from the internal camera. Alternatively, and arguably a nobler method to utilize AR is by using transparent devices which would not need the intermediate process. Heads up displays in various vehicles serve up as an early example, yet the usage of such displays is considered rather limited to primitive information. Much of the technology to build a fully transparent, independently portable gadget is yet to be realized, but the concept is no doubt an ideal that will eventually be pursued. Since every technology’s operational bounds are limited by the capacity of its power source, these emerging transparent devices will need a resembling battery which possesses similar optical properties.

In light of these rising needs, few preceding researches have attempted to fabricate such systems. Nevertheless, their operations were limited to very specific usage for *in vivo* situations^2^, or the electrochromic nature hindered true optical clarity during the charged states^3^. The first cell contraption to meet such criteria of a truly transparent, self-standing, flexible electrical energy source was developed by Yang *et al*.^4^. The lithium ion battery (LIB) secures transparency by using a patterned polydimethylsiloxane and confining the electrochemically active species in a channel which had dimensions that were smaller than the resolution of human eyes. Despite its novelty and its brilliance, setbacks were also evident not only from the intrinsic issues arising from the LIB system, but also exhibited limited battery cyclability as decreases in specific capacity is seen as early as 5 cycles. Furthermore, the arduous fabrication procedure required to construct the LIB possesses as a significant obstacle for mass scale productions.

Herein we introduce a first ever transparent, secondary zinc air battery (ZAB) which may be a method in part to overcome such issues of the prior researches. Currently, LIBs are the preferred choice of the market. However, the system’s limited energy capacity and safety issues, which are not separate as pushing for compactness comes at cost from its safety, are becoming a significant Achilles’ heel^5^. In comparison, the ZAB system withholds numerous advantages such as: high energy density, abundance of raw materials and minimal safety concerns^6–9^. These aspects have led to the selection of this particular redox pair as the development basis for the presented research. The true uniqueness of the battery is not only from its optical properties but also from its mechanical robustness. Owing from its configuration, the cell exhibits exceptional bending properties. Free bending ZABs are not unfamiliar to the scientific field, as numerous research efforts have been allocated to designing electrode and electrolyte materials for flexible ZABs^10–19^. Yet, this study encompasses a more generalized fundamental method to achieve conformational freedom along with the transparent optical properties. It should be mentioned that although optimization of the cell components is a crucial factor for high performance batteries with advanced physical features^20^, this work attempts to portray an approach that is not narrowed by the specificity of the materials but only its capability. By fabricating with different redox pairs allows the versatility and extension of the technique towards developing similar products with similar characteristics.

## Results

**Figure 1:**
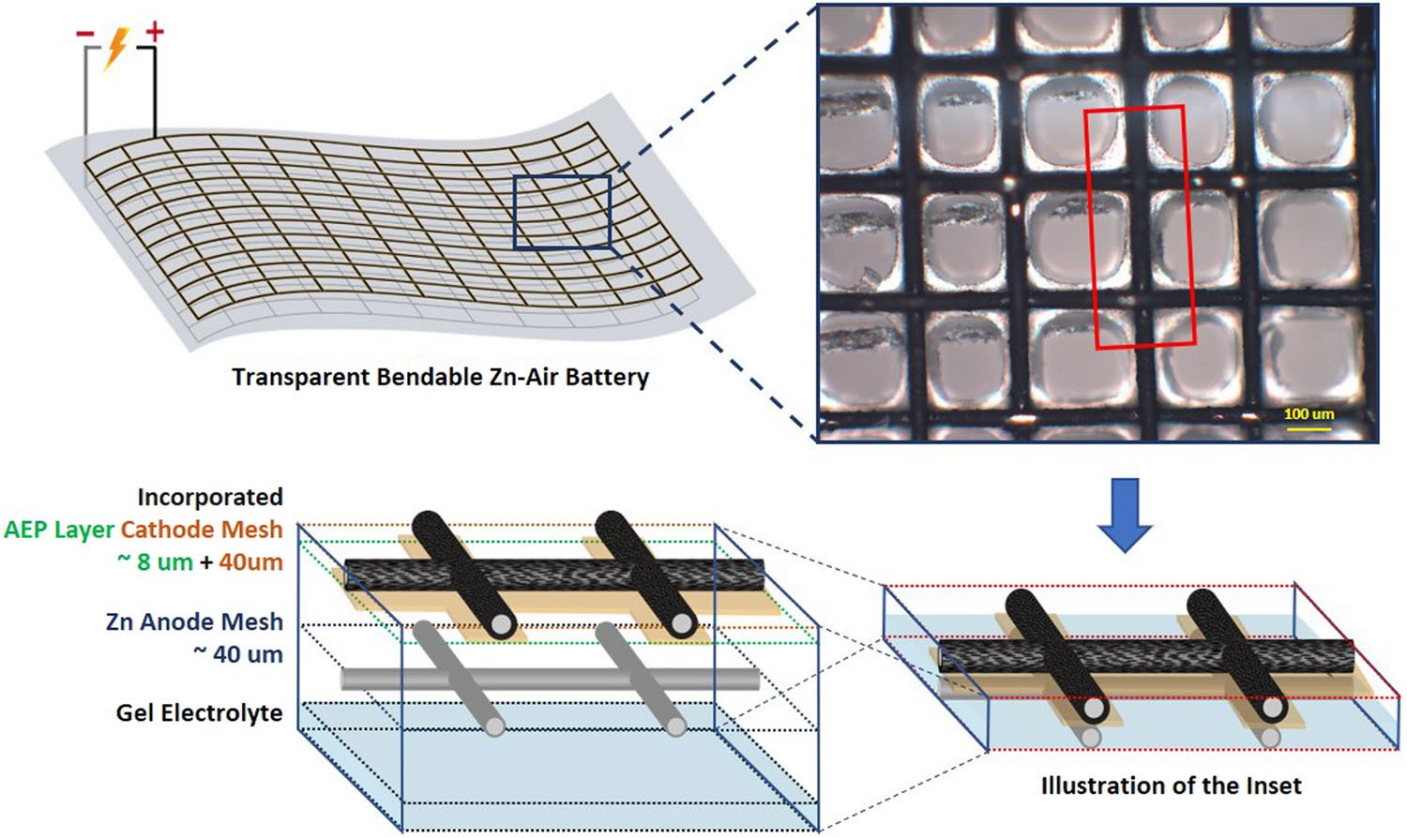
An illustrative schematic of the transparent ZAB and its components. The included optical microscope image shows the battery’s configuration as seen from the top of the cathode surface.

Figure 1 shows an illustration and the top view optical microscope image of the assembled configuration. Thickness of the battery was fabricated averagely at 90 µm disregarding the substrate film. The battery utilizes a robust, commercially available transparent stainless-steel mesh (SSM) as the current collector for both electrodes to secure its optical and mechanical properties. While numerous transparent conductors have been reported such as indium tin oxide (ITO)^21^, silver nanowire networks (Ag NW)^22^ and conductive polymers^23^, the use of these materials were inhibited because not only the conductor should be optically clear but also the redox materials utilized should maintain transparency. Conductive meshes provide well defined continuous electron passages which reduces the resistance of the cell. In this sense, in developing transparent battery systems the circumstances required the confinement of the electrochemically active species to a minimal domain to guarantee light passage while having physical durability. Here, the transparent mesh attracts application as a more reliable option compared to the inherent wide-spreading and the less mechanically durable nature of other competing current collectors (e.g., ITO, Ag NW and etc.).

Flow diagram of Supplementary Fig. S1 narrates the major procedures for constructing the battery. Two separate methods were exploited to apply the electrochemically active materials for the different electrodes. In order to produce the transparent anode, the method of electrodeposition was used. This particular technique has distinctive advantages. Firstly, it allows to densely pack the active material in a confined space closer to the current collector gaining higher energy density without significant volume growth. This is beneficial for maintaining the transparency of the system and also mitigates the effect of free diffusion for zinc ion species reducing the polarization of the cell. Comparative electrochemical impedance spectroscopy (EIS) was performed with two differently fabricated anodes to illustrate the effectiveness of the electrodeposition method (Supplementary Fig. S2). In contrast to the mentioned technique, a second anode was prepared by using a polymeric binder to coat the SSM with zinc species^14^. With the synthesized anodes, a cobalt oxide based commercial air electrode, and a 10 wt% 6 mol KOH poly acrylic acid (PAA) gel was used to create a battery setup to perform the analysis. This standardized system was introduced to examine the differential effects of the anode fabrication method as all other variables were kept constant. The spectra results revealed that the electrodeposition method has better conductivity within the electrodes as the ohmic resistance is only 73% of that of the coated anode (8.71 Ω and 11.92 Ω, respectively). Furthermore, electrodeposition is a process which is considered facile and favorable enough to extend in mass-scale levels granting this process the potential to be explored in commercial domains.

For the synthesis of the cathode, the noble metal catalysts were spray painted with a binder to the current collector. Similar approaches with SSM were made by Fu *et al*.^15^. The mesh type cathode exhibited exceptional bending properties along with an impressively stable battery operation. Yang *et al*., also demonstrated SSM substrate air catalyst cathode for Li – air batteries with brilliant electrochemical and physical characteristics^24^. A blend of commercially purchased Pt/C and Ir/C was utilized for the air electrode due to their known high activity respectively in oxygen reduction reaction (ORR)^25^ and oxygen evolution reaction (OER)^26,27^. Special attention has been given for the selection of catalyst as high volumetric specific activity was required to generate sufficient current without decreasing the visual properties. The produced cathode mesh was taken a step further to incorporate the separator.

In order to guarantee sufficient light passage while limiting any possibilities of battery shortage under conformational stress, the membrane must hold characteristics of both transparency and physical robustness. Separator-electrolyte domain has been noted as a key factor in realizing flexible durable ZABs^12,13,16^. While the majority of the developed materials with previous researches withholds extensive possibilities, much of them neglects to study their optical properties. The uniqueness of the presented work finds ground in a clever approach to address such uninvestigated areas in battery research. By exploiting micro-scale adhesion characteristics of polymers, the research provides a facile method to fabricate separators that are optically transparent and physically robust.

**Figure 2:**
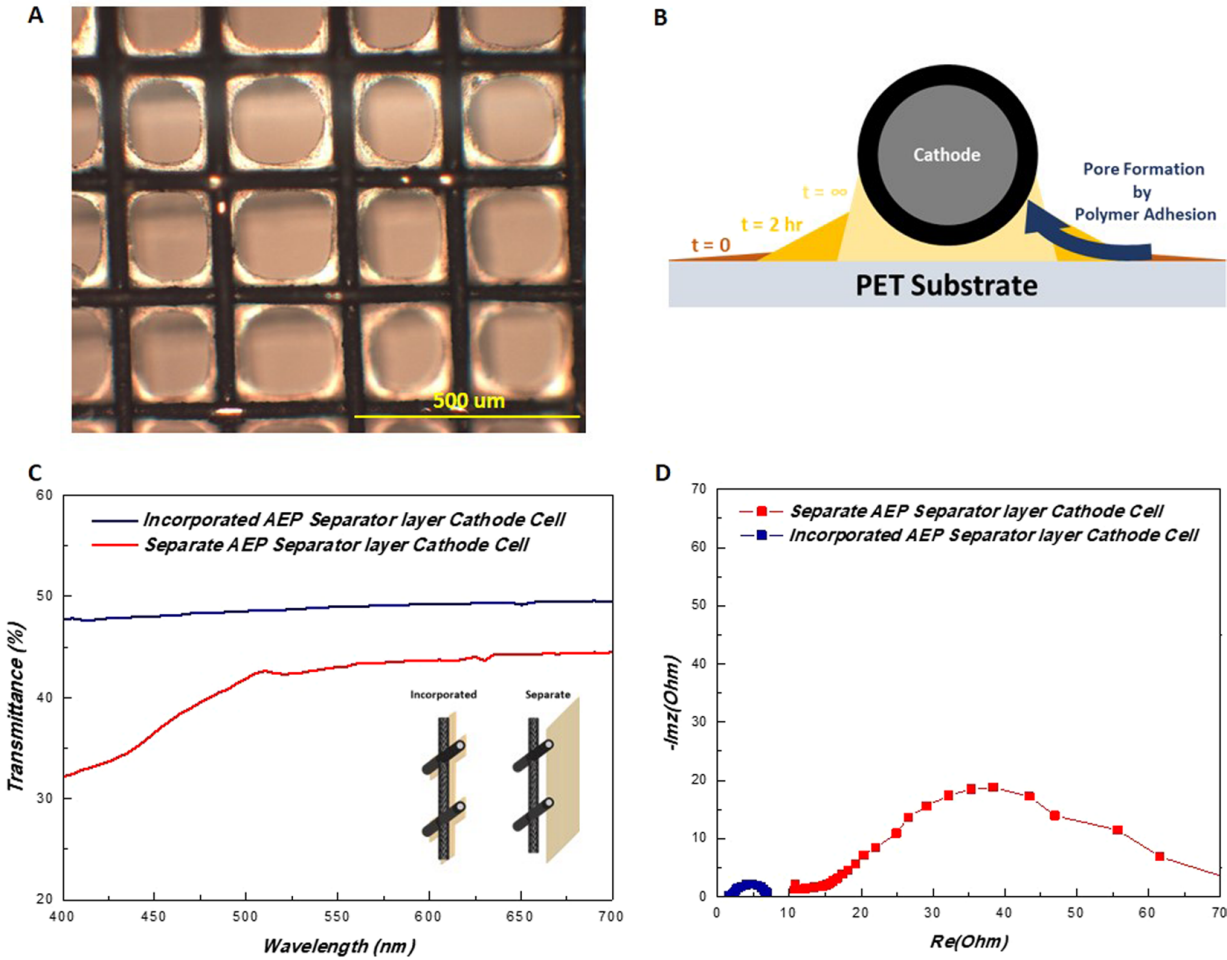
(**A**) Optical images of the cathode AEP layer pore formation. (**B**) Controlled void formation mechanism by polymer adhesion of the cathode mesh wires. Comparative (**C**) transmittance results, (**D**) EIS of the produced cell with the cathode-separator incorporated set and the separate set.

An anion exchange polymer (AEP) solution was first casted on a polyethylene terephthalate (PETE) film on top of a leveled surface. The AEP solution was maintained in an arid condition to dry partially, after which the cathode mesh was left to settle on top of the semi dried AEP. This allows the cathode and the separator to become a conjoined set. The most distinctive structure arising from this process, is the formation of selective voids in the open areas of the mesh which is shown in the optical microscope image of Fig. 2A. The formation of these controlled pores is due to the process of the polymer resin forcing to adhere to the cathode wires, resulting a selective coverage of the electrode with an electrically insulating phase. As the cathode mesh is settled on top of the AEP resin, the resin gradually seeps toward the wires leaving void spaces behind (Fig. 2B).

These evolutions of selective pores benefit in transparency of the cell as less hurdles exist in the light passage. One tradeoff that the AEP exhibits regarding its visual properties, is that the polymer taints the battery slightly yellow. AEP in general contains aromatic amine functional groups which is famed for its yellow colors^28^. Higher light absorbance in the blue region results a yellowish substance to the naked eye. Portrayed in Fig. 2C, is the transmittance data of the two fabricated cells with differing separator incorporation schemes. Utilization of the selective void separator increases general light passage for all visible regions. Yet, the most drastic disparity is seen in the violet region as the yellowness of the AEP layer decreases the blue light transmittance.

This incorporated configuration also benefits cell performance as it is possible to fabricate thinner battery systems. The thickness of the AEP layer depends on the configurational state of the wire inside the mesh. Since the structure of the mesh is similar to that of a woven fabric, the wire propagates while alternating between over and under respectively to the perpendicular wires. This results in a variance of the length of the AEP layer, which at thicker areas are measured up to 13 µm and thinner areas measure up to 3 µm averaging to 8 µm (Fig. S3. supporting Information). While such thin separators are extremely fragile in their properties, the cathode mesh acts as a reinforcement to the AEP layer granting the physical robustness of the battery. Although the equivalent circuitry of a typical ZAB will be dealt in detail in the following sections, this structure shows a decrease in both ohmic and mass transfer resistance due to its configuration (Fig. 2D). Internal ohmic resistance decreases significantly as the incorporated assembly allows thinner electrolyte/separator layers. Furthermore, the separate membrane cell requires the addition of a certain amount of electrolyte to the catalyst layer for ionic conduction. This necessity of liquid phase floods the air electrode, hindering oxygen transfer which exhibits as the larger low frequency arc. In comparison, the incorporated structure due to its thinness, only requires minimal ionic gel to be applied at the anode. This in turn forms a more pristine triple phase boundary which facilitates oxygen transfer.

One further profit of using the AEP layer comes as an improvement in cyclic stability as extensive earlier investigations revealed that the use of ion specific membranes, namely AEPs, may boost the cell’s longevity, or its cyclic stability, as it anchors zinc species migration^29–31^. Free diffusion of zincate ion leads to a certain number of degradation mechanisms. Especially when the cathode is exposed to the electrolyte saturated with zinc ions, these anions form an oxide coating which poisons the active catalyst.

For the final assembly step, anode mesh was first laid on top of a transparent PETE substrate. With the comers of the anode mesh immobilized by a gluing agent, a thin layer of 10 wt% 6 mol KOH PAA gel electrolyte was plastered with a doctor blade. After which the cathode mesh-separator assembly was placed on top of the anode mesh under a magnifying glass with extreme care to align the meshes. Displacement of alignment creates a phenomenon known as the Moiré’s pattern which is a detrimental factor for optical clearness^32^. Lastly, a gas insulating gasket with a size of 4 cm^2^ (2 × 2 cm) was used to cover the battery in order to limit the dimensions of the cell for precise characterization calculations of its performance.

## Discussion

**Figure 3:**
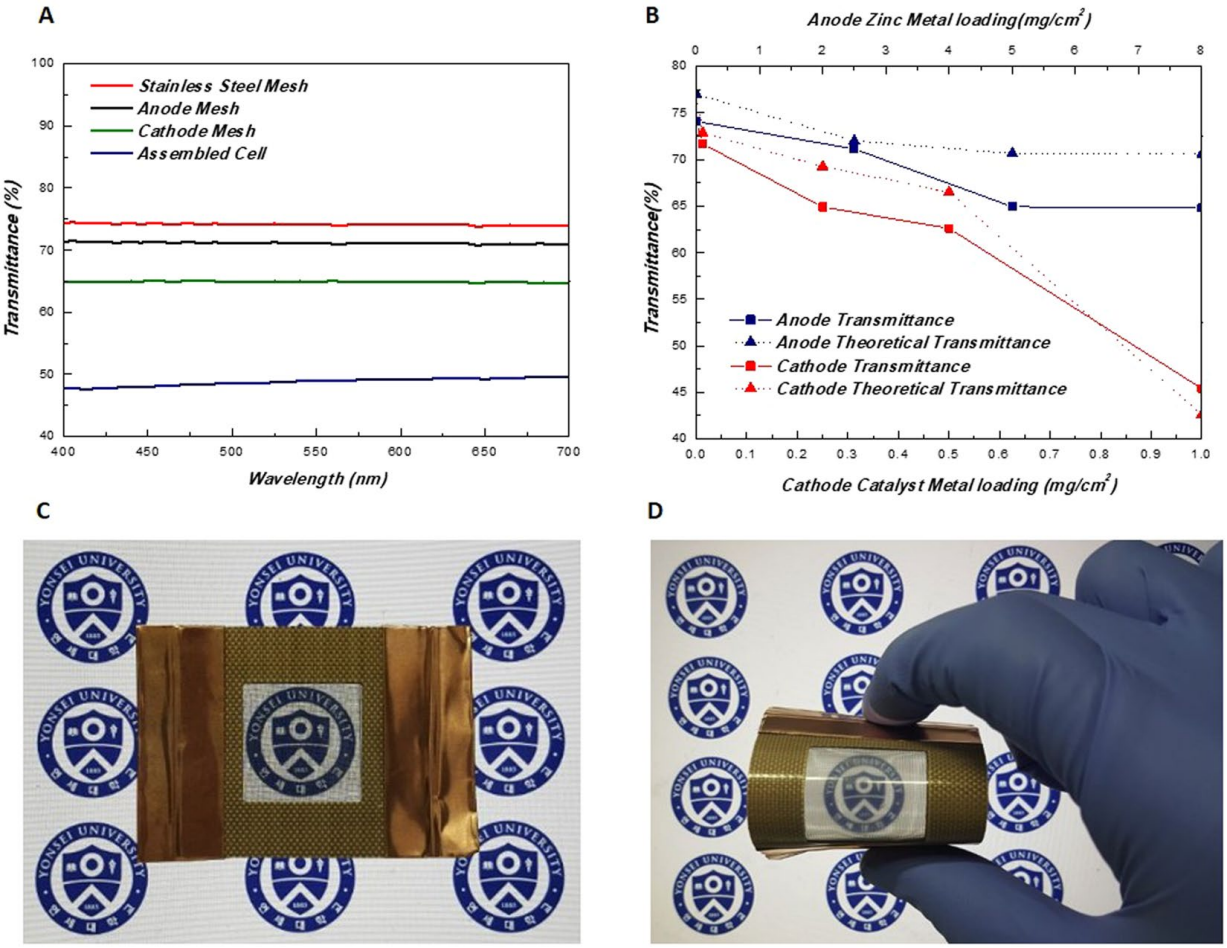
(**A**) Visible light (wavelength: 400~700 nm) transmission spectra of the components and the assembled cell. (**B**) Theoretical and actual transmittance trend graph in accordance with the dimensions of the constructed electrodes. Optical images of the cell (**C**) without bending and (**D**) with bending (image obtained with a laptop display as background).

The constructed cell’s general transparency data is shown in Fig. 3A. Since the visible wavelength of the light is throughout range of 400~700 nm, transmittance data was collected accordingly. The pristine SSM shows a transmittance average of 74.1%. Slight decrease in light passage is monitored for the produced anode and cathode as its respective average transmittance was 71.1% and 64.9%. The decrease at the cathode is slightly higher due to the thicker nature of the cathode compared with the anode. Overall cell transmittance which includes both electrodes, the gel electrolyte, the separator and the bottom PETE substrate is averaged at 48.8%. Assembly procedure and the electrolyte tariffs the transparency approximately 15~20% in relation to the electrode transmittance.

As it is evident that a clear tradeoff relation between the amount of applied redox species and light passage exists, further investigations relating dimensions and transparency were studied extensively. The results are summarized in Fig. 3B. Thickness of the meshes were measured from optical microscope images and averaged by three randomly selected points (Supplementary Fig. S4). Theoretical transmittance was also calculated assuming mesh as a 2D plane slit obstructing the incoming light rather than 3D structured object. The diagram depicting the basis of the calculation is also portrayed in Supplementary Fig. S5. Both electrodes showed a general linear decrease in transparency as the amount of electrochemical active species were increased. Furthermore, the actual transparency had a marginal deviation to the theoretical values confirming that the 2D assumption is a valid approximation of the visual properties. With these considerations the amount of anode zinc selected was 2 mg/cm^2^ and 0.25 mg/cm^2^ for the cathode catalyst. Actual light passage was monitored by the naked eye and according photos were taken (Fig. 3C,D). Transparent properties were sufficiently retained even during bended states yet slight grid like patterns were observed due to the misaligning of the electrode meshes.

**Figure 4:**
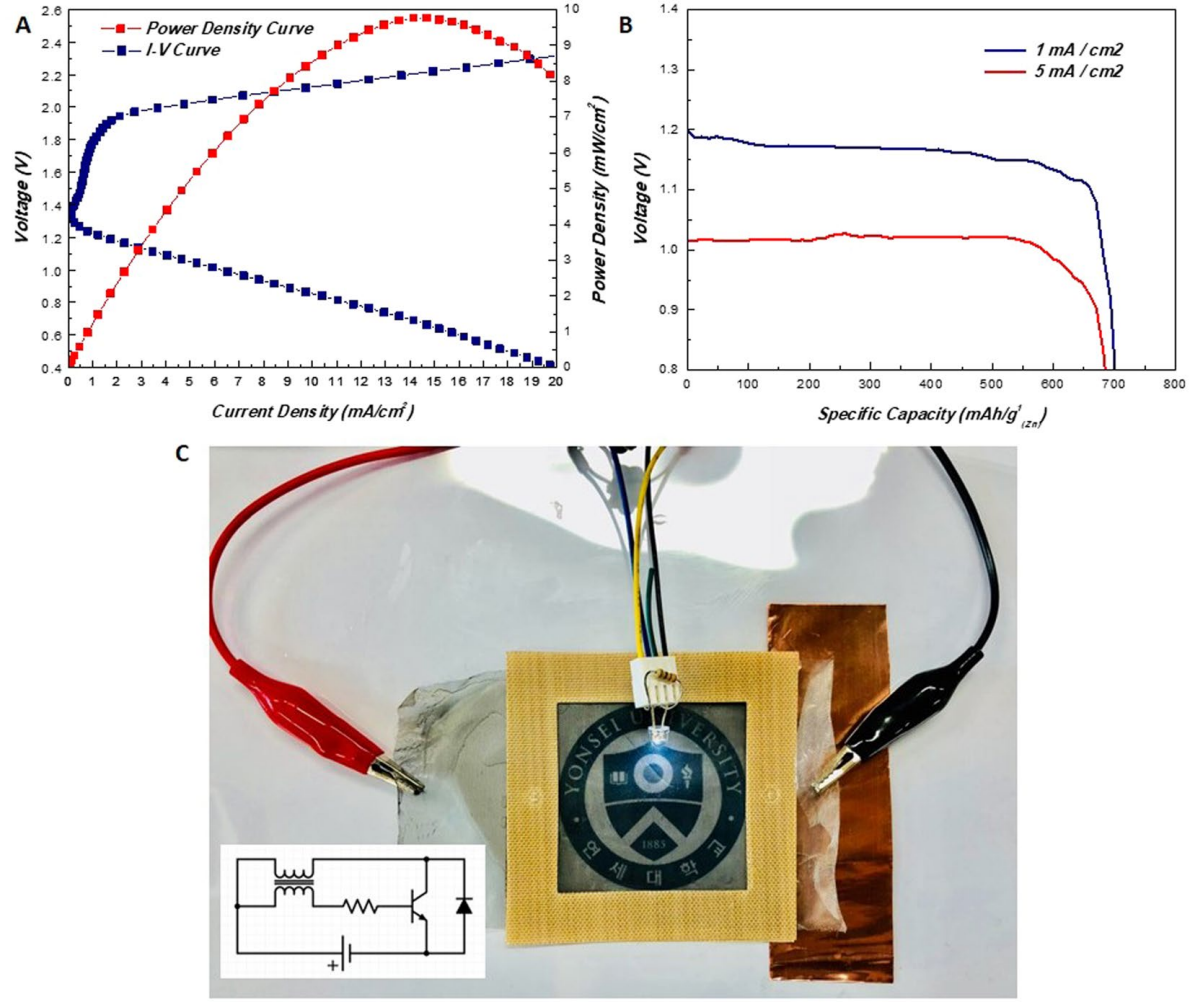
(**A**) I–V polarization/power density curves, (**B**) Galvanostatic full discharge profile of the transparent ZAB without any applied force. (**C**) Photo of a white light emitting diode (LED) powered by the transparent ZAB cell. Simple voltage transformers were used to raise the ZAB’s voltage to supply the required higher potential to light LEDs.

All electrochemical performance characterization of the cell has been studied under ambient condition and the properties were normalized to the extrinsic surface area of the battery. Initially, in order to evaluate the performance of the system, current voltage and power density curves were measured. The 1 mA/cm^2^ discharge voltage was recorded at 1.22 V and the charge voltage was recorded at 1.79 V. Maximum power density was recorded at 9.77 mW/cm^2^ (Fig. 4A). As seen in Fig. 4B, galvanostatic full discharge results revealed that the specific capacity (normalized to the amount of Zn) of the system was ~701 mAh/g_(Zn)_ and ~684 mAh/g_(Zn)_ respectively under 1 mA/cm^2^ and 5 mA/cm^2^ discharging conditions. It should be noted that the theoretical specific capacity of Zn is 820 mAh/g_(Zn)_. Actual operation of the battery was carried out by lighting a white light emitting diode (LED) with the produced transparent ZAB (Fig. 4C). In order to achieve the required minimum voltage for operating white LEDs, which is in the region of 3~5 V, a small contraption involving a voltage transformer and a transistor was used. Made with a torus permanent magnet and hand wound wiring, the transformer setup quadruples the voltage of the original cell. This allows the white LED to be powered with single alkaline batteries that has similar OCVs with the ZABs. The detailed set up is portrayed in the electrical circuit diagram in the inset.

**Figure 5:**
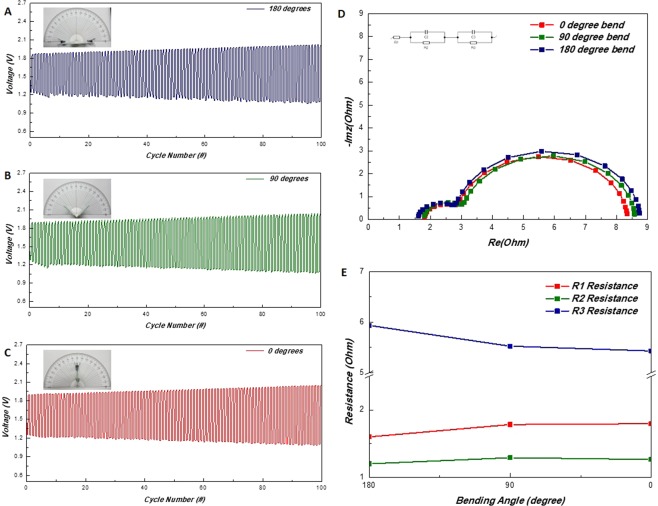
(**A**–**C**) Battery cycle results of the cell under various bending angles (180, 90 and 0°) and their corresponding states are shown in the inset photos. (**D**) Nyquist plots of the cell under the respected bending regimes and (**E**) the plotted R1, R2, R3 resistance trends.

Typical galvanostatic charge-discharge regime was employed to study the cyclic stability of the system. Each cycle consisted of 20 min discharge, charge step (overall 40 min per cycle) and the current density was maintained at 1 mA/cm^2^. Further on, to study the malleability of the system, the cell was bended with a constant force at selected angles with a contraption made with a hinged glass plate. Pins were used to hold the battery if necessary. The three angles selected for the evaluation were: 180, 90 and 0°. The bending proceeded in the manner where the cathode surface was folded outwards. Photographs of the curved battery along with their respected charge-discharge cycle test data is given in Fig. 5A–C. The initial open circuit voltage (OCV) was measured at 1.50, 1.42 and 1.44 V respectively for the tested angles. All cells exceeded to operate over 100 cycles steadily confirming the high stability of the system. Test results are summarized in the provided additional supporting information section by a table (Supplementary Table S6). While during the stable operation region of cycle (after 7 cycles), all three cells showed minimal deviation from each other, the initial voltage profile of the 90°, 180° shows a slight nonconformity. This phenomenon of initial overpotential growth was observed and studied in our previous works with similar catalyst, and was concluded such behavior arises from the activation of the cathode^29^. Typical discharge voltage at 1 mA/cm^2^ was in the range of 1.1 V to 1.2 V for all conditions, which is in accordance with the current density – voltage polarization curve.

To understand the effects of mechanical force applied to the battery, EIS was utilized. Similar equivalent circuitry has been used to interpret the internal resistance of ZABs by preceding researches (Fig. 5D inset)^14,33,34^. R1 is related to the ohmic resistances of the components and the electrolyte, R2 depicts the interfacial resistances from the electrode surface and the electrolyte and R3 is correlated to the charge transfer resistances of the cathode during reactions. Although the change in the resistance is minimal for all three resistances for respective angles, nevertheless a certain trend does appear according with the force applied. Bending the battery results in an increase of R1 resistance (1.60 Ω at 180° to 1.80 Ω at 0°) which is attributed the ohmic resistance increased by the folded current collectors. In a typical electrical conducting medium, the resistance growth for bending the current collector should be miniscule and thus be considered negligible. Yet, since the current conductor holds similar characteristics to a fabric, it is hypothesized that the folding action relaxes the tensile forces on the wires to minimize the pressure on the wire intersection increasing the resistance. In order to support this theory, with a potentiostat a comparative two-probe resistance measurement of a bare SSM was carried out. While for the bended state which had an approximate 90° angle the average resistance was measured at 2.10 Ω, for the un-bended state the average resistance was measured at 0.98 Ω. Details of the experiment are recorded in Supplementary Table S7. Concerning the R2 resistance of the battery, ideally, since the cathode – electrolyte interface has been conjoined due to the unique structure of cell, deviations should not be presented between the different states. Although the acquired spectra do exhibit some variances in the resistances (1.26 Ω at 0° to 1.20 Ω at 180°), it could be considered small enough to concur the ideality of the built. R3 decrease (5.93 Ω at 180° to 5.42 Ω at 0°) at higher angles is a result of the increased the cathode surface area due to the stretching occurring at the hinge. Similar results have been reported in polymer electrolyte fuel cell research where stretching the catalyst layer facilitates mass transport^35^. Overall, the results confirm, that the structure bestows battery the exceptional conformational freedom without tarnishing the performance.

## Conclusion

In overview, a first ever transparent, flexible, secondary ZABs were manufactured. Compared to preceding studies, due to the numerous advantages of the ZAB system, the developed cell out performs in aspects of energy density. The assembled system had an average transmittance of 48.8% in the visible region. The cell displayed a stable galvanostatic discharge-charge properties which exceeded more than 100 cycles. The maximum power density was measured at 9.77 mW/cm^2^. EIS results verified the remarkable mechanical properties of the system. The building of the battery requires industrially familiar processes which facilitates mass production. The approach also can be expanded as forerunner strategy for developing transparent batteries with other redox couples. Finally, it is important to state that the focus of the presented work has never been to fabricate ZABs with exceptional power density, nor to develop new materials that perform outstandingly. Rather the major object was to develop a facile method to realize batteries with newer physical properties which has been called upon by the emerging needs of the industry. In this sense, this approach stands as a starting point, expandable without materialistic limitations.

## Methods

### Materials

Transparent stainless-steel mesh (SSM) was purchased from a commercial vendor (100 mesh, wire diameter: 30.48 µm, opening: 223.52 µm, opening %: 79%) as well as the AEP resin (FAA-3-SOLUT-10 in NMP, FUMATECH BWT GmbH). Zinc acetate dihydrate, PAA (average M_v_ ~ 450,000), 5% Nafion solution was purchased from Sigma Aldrich and was used without further purification. Acetic acid and KOH pellets were separately obtained from Ducksan. For the electrocatalyst, a blend of 40% Pt/C (0.07 g, HISPEC 3000, Johnson Matthey Company) and 20% Ir/C (0.07 g, Premetek Co.) was used.

### Cell preparation

#### Transparent zinc anode synthesis

A simple and facile electrodeposition method was used to coat metallic zinc to the current collectors. For the electroplating bath, zinc acetate dihydrate (25 g, 98%) was fully dissolved in a 10 wt% acetic acid solution (200 ml, 99.0%), by constant stirring. The SSM was measured and cut complying to the dimensions needed for the battery and was connected to the cathode of the power supply. For the sacrificial anode, a pure zinc plate larger than the size of the SSM was used ensuring even coating. While the SSM and the zinc plate was set in a parallel configuration distancing 1 cm apart, the current applied by a power source was maintained at 250 mA cm^−2^. The total system was set in an ultrasonic bath in order to eradicate hydrogen bubbles appearing at the surface of the SSM during the initial moments of the process. The sonication was halted as soon as the hydrogen evolution was no longer visible on the surface of the mesh. The process continued until target amounts of zinc were deposited after which the produced mesh was cleansed in distilled water. The finished electrode was dried in vacuum until further usage.

#### Preparation of the transparent cathode – AEP separator assembly

For the transparent cathode, simple air brush technique was employed for the synthesis. In order to produce the catalyst slurry, Pt/C (0.07 g,) and Ir/C (0.07 g) was dispersed in isopropyl alcohol (20 ml) with 5 wt% Nafion solution as the binder (1 ml). The slurry was left to mechanically stir for 6 hrs. The same SSM was used as the current collector for the cathode and was sprayed with the catalyst ink until targeted amounts were reached. After which the electrodes were dried in an oven set at 50 °C.

For the separator of the battery, a thin layer of AEP was used. On a leveled PETE film 10% AEP solution was casted by an 80 µm doctor blade. The surface was heated to 50 °C and left to dry in ambient air for 90 min until a semi-dried state was achieved, after which the cathode mesh was settled without any external force. The assembly was left to dry further under same conditions overnight.

#### Fabrication of the transparent ZAB cell

For the structural integrity a clear PETE film was used as a substrate. The prepared zinc mesh was restrained on all four corners by a double-sided tape and a layer of 10 wt% 6 mol KOH PAA gel was casted as the electrolyte. The cathode mesh assembly was aligned and positioned over the anode under the assistance of a magnifying glass to secure maximum transparency. Finally, a gasket was placed for cell dimension restriction and copper tape was attached from the electrodes as the current leads.

### Transparent ZAB Characterization

#### Physical characterization

Visible light transmission spectra ware obtained using a UV-Vis spectrometer (Mecasys, Optizen 2120UV) in the wavelength range of 400~700 nm. Magnified images with length measurements were gained with an optical microscope (Motic, BA310MET). Field emission scanning electron microscope (FE-SEM, JSM-7800F, JEOL, Ltd) was used to visualize the cross-sectional images of the assembled batteries.

#### Electrochemical analysis

Battery cycle test was carried out on a conventional cycler system (WBCS3000, WonATech). The galvanostatic cycle consisted 1 mA, 5 min discharge-charge routine with a cutoff voltage of 0.8 V for discharge and 2.7 V for charge. In order to obtain the I-V polarization curve and the EIS spectra, multichannel potentiostat (VMP3B-10, Bio-logic) was employed. EIS results were acquired at 1.0 V vs Ref between the range of 0.1 Hz to 0.1 MHz with an alternating current amplitude of 14.14 V_rms_. All spectra were refitted by the z-fit function provided in the EC-Lab software (version 11.01).

## Supplementary information

Supplementary Information
