# Supplementary material for: Transparent Bendable Secondary Zinc-Air Batteries by Controlled Void Ionic Separators
Source: Sci Rep. 2019 Feb 28;9:3175. doi: 10.1038/s41598-019-38552-4 (PMC6395654; doi:10.1038/s41598-019-38552-4)
Supplement: Supplementary file 1 — Supplementary Information [file 41598_2019_38552_MOESM1_ESM.pdf]

Supporting Information

Transparent Bendable Secondary Zinc Air Batteries by Controlled Void Ionic Separators

Ohchan Kwon[1], Ho Jung Hwang[2], Yunseong Ji[1], Ok Sung Jeon[1], Jeong Pil Kim[1], Chanmin Lee[1] and Yong Gun Shul[1,2]*.*

[1]Department of Chemical and Biomolecular Engineering, Yonsei University,262 Seongsanno, Seodaemun-gu, Seoul 120-749, South Korea

[2]New energy and battery engineering, Yonsei University, 134 Shinchon-dong, Seodaemoon-ku, Seoul 120-749, Republic of Korea

S1. Narrative flow diagram of the fabricating procedure of the transparent zinc air battery

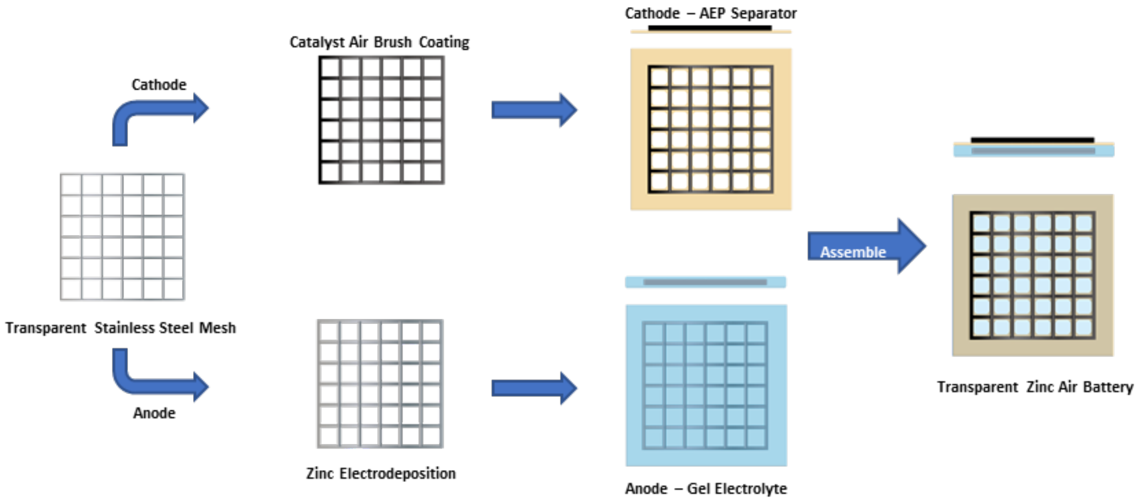

S2. Ohmic resistances of the electrodeposited anode and the coated anode. The polymer used for binding the zinc powder is PVDF-HFP. In order to maintain consistency, other components were kept constant with an aqueous solution of 6 mol KOH as the electrolyte and a commercial Co oxide-based air electrode as the cathode.

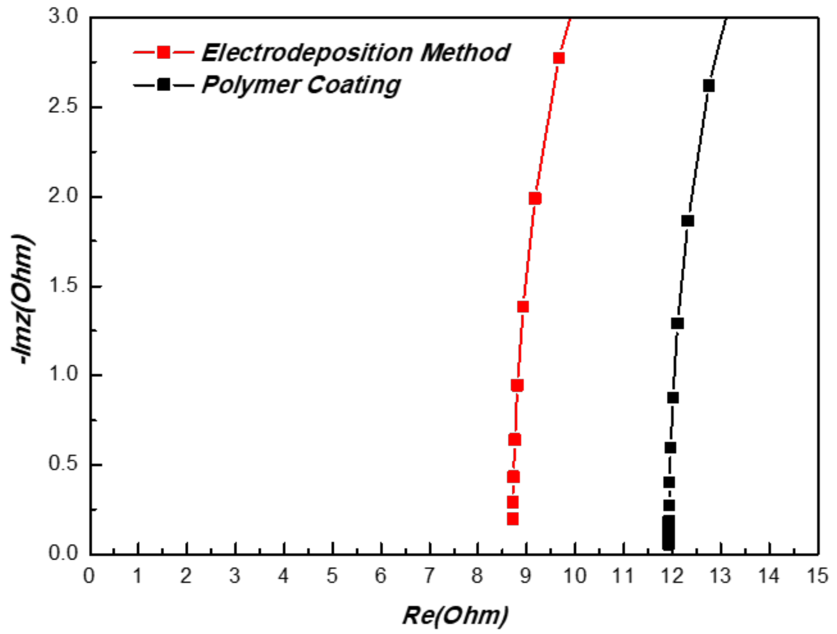

S3. Cross sectional SEM images of the cathode-separator conjoined set. Due to the fabric like structure of the mesh, the thickness of the AEP applied varies as some wires are woven over and some are woven under. Average thickness of the separator is estimated to be around 8um.

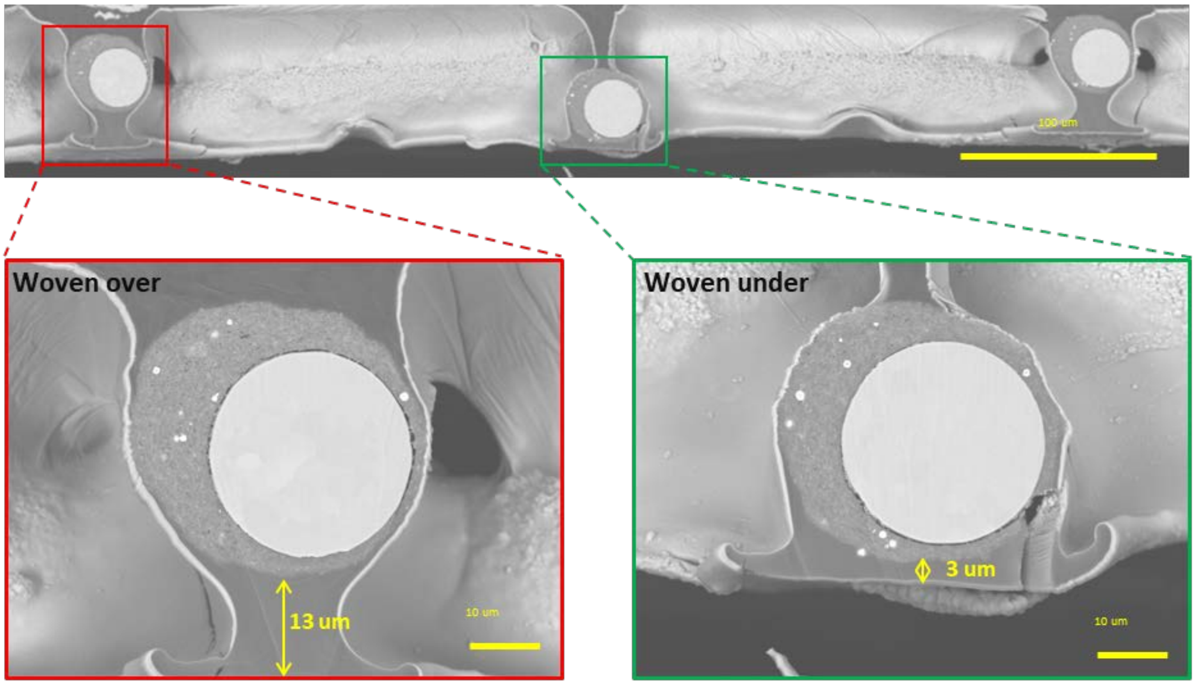

S4. Optical microscope images of the pristine SS mesh and the electrodes with varying redox material loading.

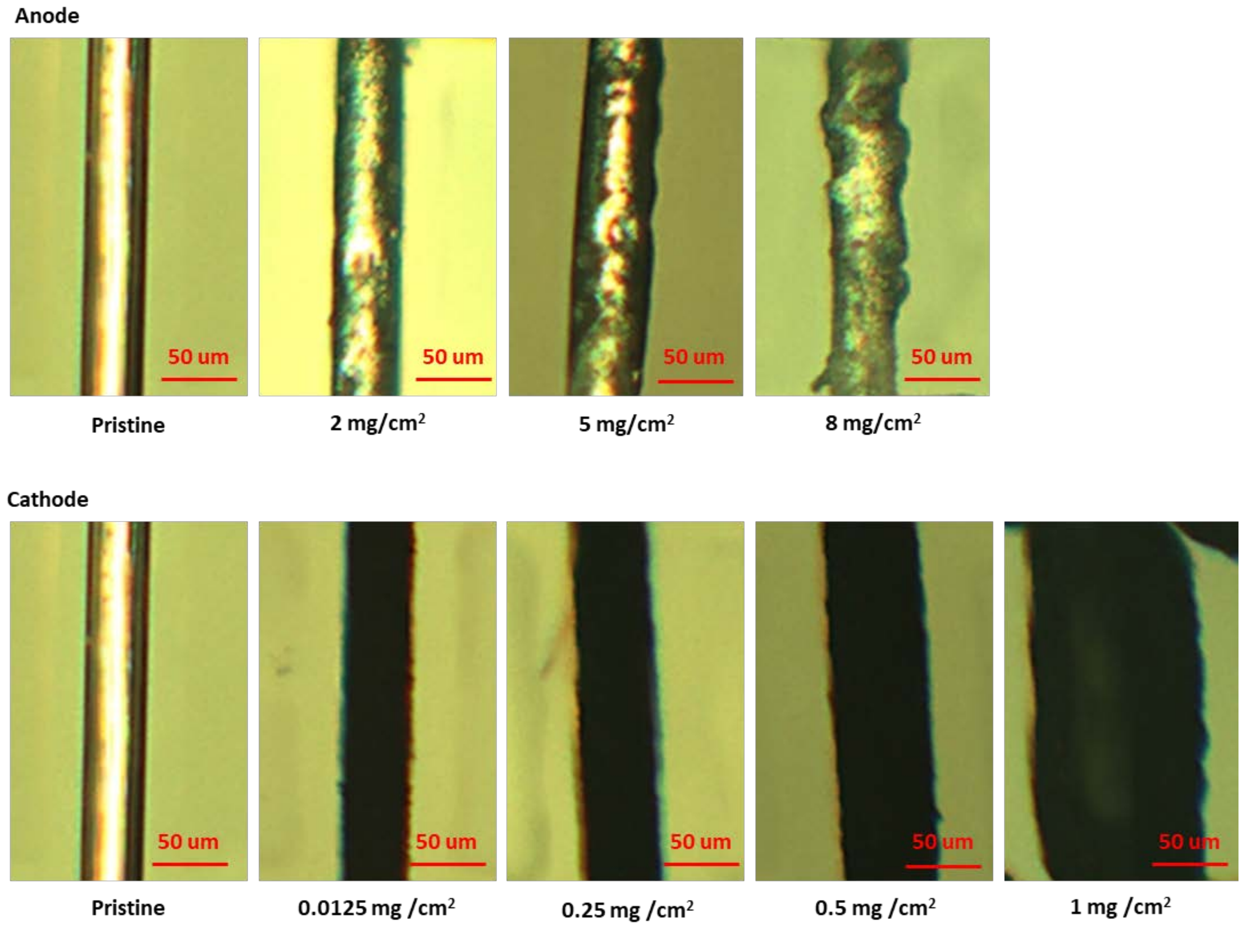

S5. Schematic diagram and the relating equation for the theoretical transparency analysis.

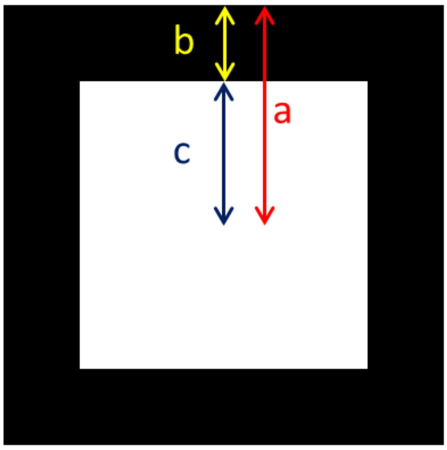

$$Transmittence\ of\ Light(\%) = \frac{Unobstructed\ Area}{Total\ Area} \times 100 = \frac{4c^2}{4a^2} \times 100 = \frac{(a-b)^2}{a^2} \times 100$$

S6. Summary of the battery cycle test operation results.

Type	OCV	Current	Time	Cut off V	Cycle #
0 degrees	1.44 V	1 mA/cm2	5 min dis/chr	0.8V/2.3V	111
90 degrees	1.42 V	1 mA/cm2	5 min dis/chr	0.8V/2.3V	106
180 degrees	1.50 V	1 mA/cm2	5 min dis/chr	0.8V/2.3V	113

S7. Resistance measurement results for the SSM during bended and un-bended states.

Resistance (Ohms)	count 1	count 2	count 3	count 4	count 5	count 6	count 7	count 8	count 9	count 10	Average
**90-degree bend**	1.84	2.21	2.00	2.53	2.03	2.58	1.95	2.05	1.85	2.01	**2.10**
**Unbended**	1.07	1.00	1.04	1.00	1.02	0.87	0.91	0.88	1.01	0.98	**0.98**

Supplementary Information Legend

Supplementary Figure S1. Narrative flow diagram of the fabricating procedure of the transparent zinc air battery

Supplementary Figure S2. Ohmic resistances of the electrodeposited anode and the coated anode. The polymer used for binding the zinc powder is PVDF-HFP. In order to maintain consistency, other components were kept constant with an aqueous solution of 6 mol KOH as the electrolyte and a commercial Co oxide-based air electrode as the cathode.

Supplementary Figure S3. Cross sectional SEM images of the cathode-separator conjoined set. Due to the fabric like structure of the mesh, the thickness of the AEP applied varies as some wires are woven over and some are woven under. Average thickness of the separator is estimated to be around 8um.

Supplementary Figure S4. Optical microscope images of the pristine SS mesh and the electrodes with varying redox material loading.

Supplementary Figure S5. Schematic diagram and the relating equation for the theoretical transparency analysis.

Supplementary Table S6. Summary of the battery cycle test operation results.

Supplementary Table S7. Resistance measurement results for the SSM during bended and un-bended states.
